# The role of beneficial bacteria wall elasticity in regulating innate immune response

**DOI:** 10.1186/s13167-015-0035-1

**Published:** 2015-06-19

**Authors:** Viktoria V. Мokrozub, Liudmyla M. Lazarenko, Liubov M. Sichel, Lidia P. Babenko, Petro M. Lytvyn, Olga M. Demchenko, Yulia O. Melnichenko, Nadiya V. Boyko, Bruno Biavati, Diana DiGioia, Rostyslav V. Bubnov, Mykola Ya Spivak

**Affiliations:** Zabolotny Institute of Microbiology and Virology, National Academy of Sciences of Ukraine, 154, Zabolotny st., Kyiv, D03680 Ukraine; Pure Research Products, LLC, 6107, Chelsea Manor Court, Boulder, CO 80301 USA; Lashkaryov Institute of Semiconductor Physics, National Academy of Sciences of Ukraine, 41, pr. Nauky, Kyiv, 03028 Ukraine; LCL «Diaprof», Svitlycky Str., 35, Kyiv, 04123 Ukraine; Dipartimento di Scienze Agrarie, Alma Mater Studiorum—Bologna University, Bologna, 40127 Italy; Clinical Hospital “Pheophania” of State Affairs Department, Zabolotny str., 21, Kyiv, 03680 Ukraine

**Keywords:** Predictive, preventive, and personalized medicine, Gut microbiota, *Lactobacillus*, *Bifidobacterium*, Cytokines, Oxidative, nitrosative stress, Macrophages, Cell wall elasticity, Atomic force microscopy, Gut–brain axis, Microbiome, Cancer, Immune disorders, Dietary biomarkers, Healthy diet, Nutrition, Fecal microbiota transplantation, Pattern-recognition receptors

## Abstract

**Background:**

Probiotics have great potential to contribute to development of healthy dietary regimes, preventive care, and an integrated approach to immunity-related disease management. The bacterial wall is a dynamic entity, depending on many components and playing an essential role in modulating immune response. The impact of cell wall elasticity on the beneficial effects of probiotic strains has not been sufficiently studied.

The aim was to investigate the effect of lactic acid bacteria (LAB) and bifidobacteria strains on phagocytic system cells (macrophages) as related to bacterial wall elasticity, estimated using atomic force microscopy (AFM).

**Methods:**

We conducted studies on Balb/c line mice 18–20 g in weight using lyophilized strains of LAB—*Lactobacillus acidophilus* IMV B-7279, *Lactobacillus casei IMV B-7280*, *Lactobacillus delbrueckii* subsp. *bulgaricus IMV B-7281*, and bifidobacteria—*Bifidobacterium animalis VKL* and *Bifidobacterium animalis VKB*. We cultivated the macrophages obtained from the peritoneal cavity of mice individually with the strains of LAB and bifidobacteria and evaluated their effect on macrophages, oxygen-dependent bactericidal activity, nitric oxide production, and immunoregulatory cytokines. We used AFM scanning to estimate bacterial cell wall elasticity.

**Results:**

All strains had a stimulating effect on the functional activity of macrophages and ability to produce NO/NO_2_ in vitro. Lactobacilli strains increased the production of IL-12 and IFN-γ in vitro. The AFM demonstrated different cell wall elasticity levels in various strains of LAB and bifidobacteria. The rigidity of the cell walls among lactobacilli was distributed as follows: *Lactobacillus acidophilus* IMV B-7279 > *Lactobacillus casei* IMV B-7280 > *Lactobacillus delbrueckii* subsp. *bulgaricus* IMV B-7281; among the strains of bifidobacteria: *B. animalis* VKB > *B. animalis* VKL. Probiotic strain survival in the macrophages depended on the bacterial cell wall elasticity and on the time of their joint cultivation.

**Conclusion:**

LAB and bifidobacteria strains stimulate immune-modulatory cytokines and active oxygen and nitrogen oxide compound production in macrophages. Strains with a more elastic cell wall according to AFM data demonstrated higher resistance to intracellular digestion in macrophages and higher level of their activation.

AFM might be considered as a fast and accurate method to assess parameters of probiotic strain cell wall to predict their immune-modulatory properties.

## Overview

The gut microbiota can be considered an extension of the self and, together with the genetic makeup, determines the physiology and metabolism of an organism [1]. Strachan [2] described the hygiene hypothesis that refers to an originally associated reduced microbial contact to microbes in early life and is suggested to be one of the main mechanisms to account for the increasing prevalence of allergic diseases over the past few decades.

Today, reduced microbial exposures (and the rise in allergic conditions) have been attributed to the healthier benefits of Western lifestyle factors such as diet, antibiotic use, vaccinations, reduced household size and improved hygiene, and low infant mortality [3]. The hygiene hypothesis states that allergy and increased longevity are both consequences of reducing infectious stressors during early childhood for millennia. Mechanistic explanations for the hygiene hypothesis have typically invoked the *Th1/Th2 model for helper T cells* and so-сalled *counter-regulatory* model that might also explain the increasing incidence of autoimmune disease in westernized countries [4].

Lactic acid bacteria (lactobacilli, LAB) inhibit the colonization of pathogenic bacteria upon the intestinal epithelium by the competition for the adherence sites on the intestinal mucosa between pathogens and lactobacilli and by the production of inhibitory substances. The host specificity of the lactobacilli is closely connected with the presence of the specific molecules of receptors on the host cells which can be distinguished by means of specific molecules of the bacterial cells [5]. Information on the cell envelope constituents of lactobacilli that influence bacterial aggregation and adhesion to intestinal cells will help to understand better their specific contribution in commensal–host interactions and adaptation [6].

### Probiotic bacterial cell wall heterogeneity—the challenge of understanding host–bacteria interaction and their potential as biomarkers

As the LAB are Gram-positive bacteria, their cell walls comprise a complex mixture of glycolipids, lipoproteins, and phosphorylated polysaccharides embedded in a thick layer of PGN, a polymer of β(1–4)-linked *N*-acetylglucosamine and *N*-acetylmuramic acid, cross-linked by short peptides [7]. The cytoplasmic membrane of Gram-positive bacteria, which constitute the vast majority of commercially applied probiotics, is covered by a thick cell wall consisting of multiple layers of peptidoglycan, capsular polysaccharide (CPS), lipoproteins, and teichoic acids. Some of these molecules contain specific *microbe-associated molecular patterns (MAMPs)* that are recognized by specific *pattern-recognition receptors (PRRs)* expressed in host intestinal mucosa [8].

*Peptidoglycan (PG)* is the largest component of the bacterial cell wall and is an essential polymer in lactobacilli that determines the shape and preserves the integrity of the bacterial cell.

The *elastic nature of PG* helps withstand stretching forces caused by bacterial turgor pressure, excludes large molecules from entering the bacterial cell, and at the same time restricts secretion of large proteins. The PG layer has been described as a “fisherman’s net”, functioning both as a container for and a sieve to the bacteria [9].

*Proteins* with theoretical molecular mass as large as 49.4 and 82.1 kDa have been reported to be secreted by *Lactobacillus rhamnosus* GG and *Lactobacillus plantarum*, respectively [10, 11].

Several studies provide evidence regarding cell envelope constituents of lactobacilli that influence bacterial *aggregation* and *adhesion* to intestinal cells [6, 12, 13]. The adhesion and aggregation ability of LAB is possessed by specific surface proteins and *fatty acid* content; thus, adherence of *L. rhamnosus* was dependent on specific interactions and was promoted by surface proteins and specific fatty acids. Polysaccharides likely hindered bacterial adhesion and aggregation by masking protein receptors [6, 12].

Peptidoglycan is partly responsible for the T helper type 1 skewing effect of certain LAB. Dendritic cells (DCs) that are TLR2^−/−^ produce less IL-12 and TNF-alpha and more IL-10 in response to some strains of lactobacilli, while they produce more IL-12 and less IL-10 in response to bifidobacteria. Cell wall fractions of all LAB as well as synthetic lipoproteins act as immunoregulators through interaction of lipoprotein with TLR2 and as immunostimulators through interaction of peptidoglycan with NOD2. Independently of the cytokine pattern induced by intact LAB, cell wall fractions of all LAB as well as synthetic lipoproteins possess immunoinhibitory capacities in DCs [7].

The ability of peptidoglycan and other non-acylated cell wall components to induce TLR2 signaling was studied [14–18]. According to study by Volz et al., contamination of peptidoglycan preparations with lipoproteins is a confounding factor [16]. *TLR2* preferentially recognizes peptidoglycan fragments containing *diaminopimelic acid*, but also recognizes *lysine-containing peptidoglycan fragments*, albeit with lower affinity [17, 18].

Moreover, the domain structure of peptidoglycan *hydrolases* offers some potential for enzyme engineering, since the combination of catalytic and CWB domains of different origin could lead in novel enzyme molecules with many desirable features. However, common use of these enzymes and finding answers to many more research questions will require more studies [19].

*Exopolysaccharides* (EPS) produced by probiotic strains modify the adhesion of probiotics and enteropathogens to human intestinal mucus [20].

Some large proteins are unable to diffuse through the cell wall and are dependent on the cell wall expansion process to be dragged to the outer surface of the thick PG layer before being passively released into the external milieu [21]. The threads of this net are polymers of covalently linked alternating residues of *N*-acetyl-glucosamine (*GlcNAc*) and β-1-4-linked *N*-acetyl-muramic acid (*MurNAc*).

The availability of full genome sequences and predicted proteomes, in combination with sophisticated genetic tools, allows the in silico identification of *microbe-associated molecular patterns* (*MAMPs*) and their validation by mutagenesis [22].

The glycan strands are held together by cross-linking pentapeptide side chains providing elasticity to the net [9, 20]. Following biosynthesis, assembly, and incorporation of the PG subunits, modifications in the GlcNAc and MurNAc structures can occur and affect interactions between host and lactobacilli [23]. These modifications can affect the physiology of the bacterial cell wall by increased sensitivity to autolysis, resistance to lysozyme, and hydrophobicity of the cell envelope which in turn affects recognition by host receptors and bacterial adhesion [21].

*Factors controlling PG hydrolysis.* A strong PG mesh is needed to maintain cell shape and to counteract both high turgor pressure and cell wall stress related to environmental factors. At the same time, the growth and separation of bacterial cells also require a high degree of PG elasticity. These two opposing demands require the coordinated and balanced action of PG synthetic and degradation enzymes [21].

PG and PGH as mediators of bacteria–host interactions PG and certain of its fragments are known as MAMPs that are recognized by host pattern-recognition receptors (PRRs), such as NOD receptors or Toll-like receptors (TLR) [24].

*Nucleotide-binding oligomerization domain receptors (NOD-like receptors, NLRs)* are intracellular receptors expressed by both epithelial cells and immune cells, such as *dendritic cells* [25–28].

Lactobacilli display considerable variation in their cell surface properties, through adaptations which undoubtedly are important for the functioning and survival of these bacteria in the gastrointestinal tract (GIT). The cell wall is a dynamic entity and plays an essential role in many aspects of the physiology and functioning of lactobacilli. Environmental stressors have been shown to affect the cell surface architecture by influencing PG biosynthesis, expression of EPS and cell surface proteins, and LTA decoration with d-alanine residues [21].

Genomics-based approaches and mutant analyses is promising to identification of several effector molecules of lactobacilli wall, involved in direct interactions with host epithelial or immune cells, and many of these effector molecules are components of the cell wall itself [29].

Considering the complexity of host–lactobacilli interactions involving host-cell signaling and regulation pathways, it seems unlikely that single-effector molecules regulate the entire host response and probably play crucial roles as building blocks of the bacterial cell wall [30].

*Cell wall hydrolases* act autonomously or on neighboring cells to modulate invasion of prey cells, cell shape, innate immune detection, intercellular communication, and competitor lysis. The hydrolases involved in these processes catalyze the cleavage of bonds throughout the sugar and peptide moities of peptidoglycan. Phenotypes associated with these diverse hydrolases reveal new functions of the bacterial cell wall beyond growth and division [31].

The probiotics are cultures of potentially beneficial bacteria that positively affect hosts by enhancing the microbial balance and therefore restore the normal intestinal permeability and gut microecology, improve the intestine’s immunologic barrier function, and reduce the generation of proinflammatory cytokine characteristics of allergic inflammation as food allergy, atopic dermatitis, and in primary prevention of atopy [3, 32].

The principle of the directed immune correction with probiotics is primarily based on the fact that the structural components of cell walls of lactic acid bacteria and bifidobacteria have an ability to interact with the receptors of pattern recognition, in particular with *TLR-receptors*, which are present on the surface of phagocytic system cells, intraepithelial T-lymphocytes, natural killer cells, etc. This leads to activation of intracellular molecular cascades, which stimulate the expression of many genes of immune response; the result of this stimulation is the production of different immunoregulatory cytokines, depending on the type of activated TLR-receptors [33, 34].

Various strains of lactic acid bacteria and bifidobacteria are able to interact with different types of TLR-receptors and therefore induce cytokine synthesis of various opposition groups, for example Th1- and Th2-types, pro- and anti-inflammatory [35, 36]. A possibility to regulate the balance between Th1/Th2 ways of immune response appears due to the purposeful influence on that or those cytokine production. Choice of Th1- or Th2-type of immune response depends on many factors, among which in the first place is pointed out is the activity of *antigen-presenting cells* (APC) and the profile of cytokines, which are synthesized in the early stage of the immune response [33, 37]. In particular, the production of IL-12 by activated macrophages will promote the differentiation of Тh0-cells in the Th1 lymphocytes and induce the development of cellular immunity, which plays a key role in protecting the body against many common infections [37]. Knowledge of the molecular mechanisms underlying the physiological characteristics of LAB and the identification and verification of effector molecules, complemented by parallel research to their respective receptors in host cells, may enhance the concept of strain specificity and contributes to the development of strains with improved health benefits [21].

As far as phagocytes, cells of the innate immunity are the first line of host defense, and they are key to protect the gut mucosa; it is important to investigate the action of probiotic bacteria in vitro on the phagocytic system cell activity and the production of immune regulatory cytokines.

It is necessary to take into account the fact that immunomodulating activity of strains can depend on the degree of their cell wall elasticity, which causes the cell’s resistance to the intracellular digestion in the macrophages and influences the level of phagocytosis completeness [38].

### Atomic force microscopy

Several imaging modalities exist for measuring elasticity of materials and tissues in macroscale like sonoelastography (ultrasound), magnetic resonance elastography, however in micro- and nanoimaging we still lack such reliable methods. Considering complexity of cell wall a simple and unified method of assessment its properties in regard to the beneficial properties is highly required in further research and clinical sets.

Atomic force microscopy is a novel method for high-resolution imaging of any surface including those of living and fixed cells. This powerful technique is also used for characterization of the mechanical, electrical and magnetic characteristics of samples to be studied both qualitatively and quantitatively [39, 40]. The elasticity of the bacterial cell wall can be estimated by nanoindentation that means, implemented the atomic force microscopy (AFM) on the basis of the method of force spectroscopy [39].

Schaer-Zammarett et al. [41] studied molecular interactions of lactic acid bacteria at the nanometer scale using AFM under native conditions to observe the surface topography of bacteria from the species *Lactobacillus crispatus*, *Lactobacillus helveticus*, *and Lactobacillus johnsonii*. Schaer-Zammaretti and Ubbink found cell wall heterogeneities caused by variations in the dominant surface constituents like proteins and polysaccharides showed major differences between bacteria having a crystalline-like protein layer as part of the cell wall. Force volume images calculated into elasticity and adhesion force maps of different bacterial strains showed that *L. crispatus and L. helveticus* have a surface with a homogeneous stiffness with no adhesion events [41].

The reliable measurements and analysis of wide variety in surface properties of probiotic bacteria might have wide-ranging implications for food processing and for health benefits.

The aim of this study was to determine the effect of probiotic strains, e.g., *Lactobacillus delbruеckii* subsp. *bulgaricus* IMV B-7281, *Lactobacillus acidophilus* IMV B-7279, *Lactobacillus casei* IMV B-7280, *Bifidobacterium animalis* VKL, and *Bifidobacterium animalis* VKB on phagocytic system cells’ functional activity in vitro and immunoregulatory cytokine synthesis, as related to the bacteria surface properties evaluated by the cell wall elasticity using atomic force microscopy.

## Methods

We performed studies on Balb/c line mice 18–20 g in weight, obtained from the vivarium of the Institute of Molecular Biology and Genetics, NAS of Ukraine. All studies were performed taking into account the rules of the European Convention for the protection of vertebrate animals used for research and other scientific purposes from 18 March 1986 and the law of Ukraine No 3447-IV “About animals protection from cruel treatment” [42].

Lyophilized strains of lactobacilli—*L. acidophilus* IMV B-7279, *L. casei* IMV B-7280, *L. delbrueckii* subsp. *bulgaricus* IMV B-7281, and bifidobacteria—*B. animalis* VKL, and *B. animalis* VKB were used. The viability of probiotic cultures was determined by monitoring their growth on the Man–Rogosa–Sharpe (MRS) nutrient medium at 37 °C for 24–48 h.

We used scanning AFM (NanoScope IIIa, Digital Instruments Inc.) to determine elasticity of the bacterial cell walls. The studied strains were recovered and synchronized on MRS nutrient medium and MRS medium 05 % of cysteine addition for lactic acid bacteria and bifidobacteria, respectively, at 37°С by 10–15 times of reseeding. Ten to 12-h cultures (the beginning of the stationary phase of growth), pre-washed with distilled water from the remnants of the cultural medium by centrifugation at 1500 rpm for 5 min, were used in the study. Cell suspension was put on the surface of fresh cleaved mica. AFM mapping of the sample’s surface was performed in the mode of periodic contact after the excess moisture removal. This mode provides a high spatial resolution of measurements and minimal deformation of the studied bacteria surface under the probe. The elasticity of the bacterial cell wall was estimated by *nanoindentation* means, implemented in the AFM on the basis of the method of force spectroscopy [43].

Macrophages, received from the peritoneal cavity of mice by common method, were cultivated with the strains of lactic acid bacteria and bifidobacteria (each of them individually) during 30, 60, 90, and 120 min at 37 °C in an atmosphere of 5 % of CO_2_ in the 1:100 cell ratio in 199 culture media with addition of 5 % of embryonic calf serum. After that, macrophages were washed from non-phagocytized bacteria and destroyed with 0.25 % solution of sodium dodecyl sulfate (Sigma, USA). The received lysates were plated on a MRS agar solution and MRS agar with the addition of 0.05 % of cysteine and grew at 37 °C in an atmosphere of 5 % of CO_2_. After 48 h of incubation, the number of colony forming units (CFU) was counted, taking into account that one colony corresponds to one bacterium.

The impact of lactic acid bacteria and bifidobacteria on the functional activity of peritoneal cavity macrophages was estimated using the conventional methods of oxygen-dependent bactericidal activity test and nitric oxide production. Their effect on the immunoregulatory cytokines production—interleukin-12 (IL-12) and interferon-γ (IFN-γ) was also determined. Macrophages were cultivated with lactic acid bacteria or bifidobacteria (each strain separately) in the 1:100 cell ratio for 24 h at 37 °C in an atmosphere of 5 % CO_2_.

The concentration of nitric oxide in the culture medium of macrophages after a 24-h cultivation with lyophilized bacteria was determined by colometric method with the Griess reagent use [44]. Oxygen-dependent bactericidal activity of macrophages was evaluated by spectrophotometric method in a nitroblue tetrazolium recovery test (NBT-test) [45].

The concentration of IFN-γ and IL-12 in the cultural medium of the macrophages was determined by enzyme immunoassay using the appropriate test systems (Bender MedSystems, Austria).

All digital data received were processed with the help of the Origin Pro software (version 8.5) through analysis of variance. Numerical data were represented as arithmetic average and standard error (Mm). The null hypothesis for the control and experimental comparative groups was checked using Wilcoxon–Mann–Whitney (*U*) and Kolmogorov–Smirnov nonparametric criteria. The differences between the groups were considered statistically meaningful at *P* < 0.05.

## Results

The analysis of the data obtained with the AFM demonstrated different degrees of the cell wall’s elasticity in various strains of lactic acid bacteria and bifidobacteria.

**Fig. 1:**
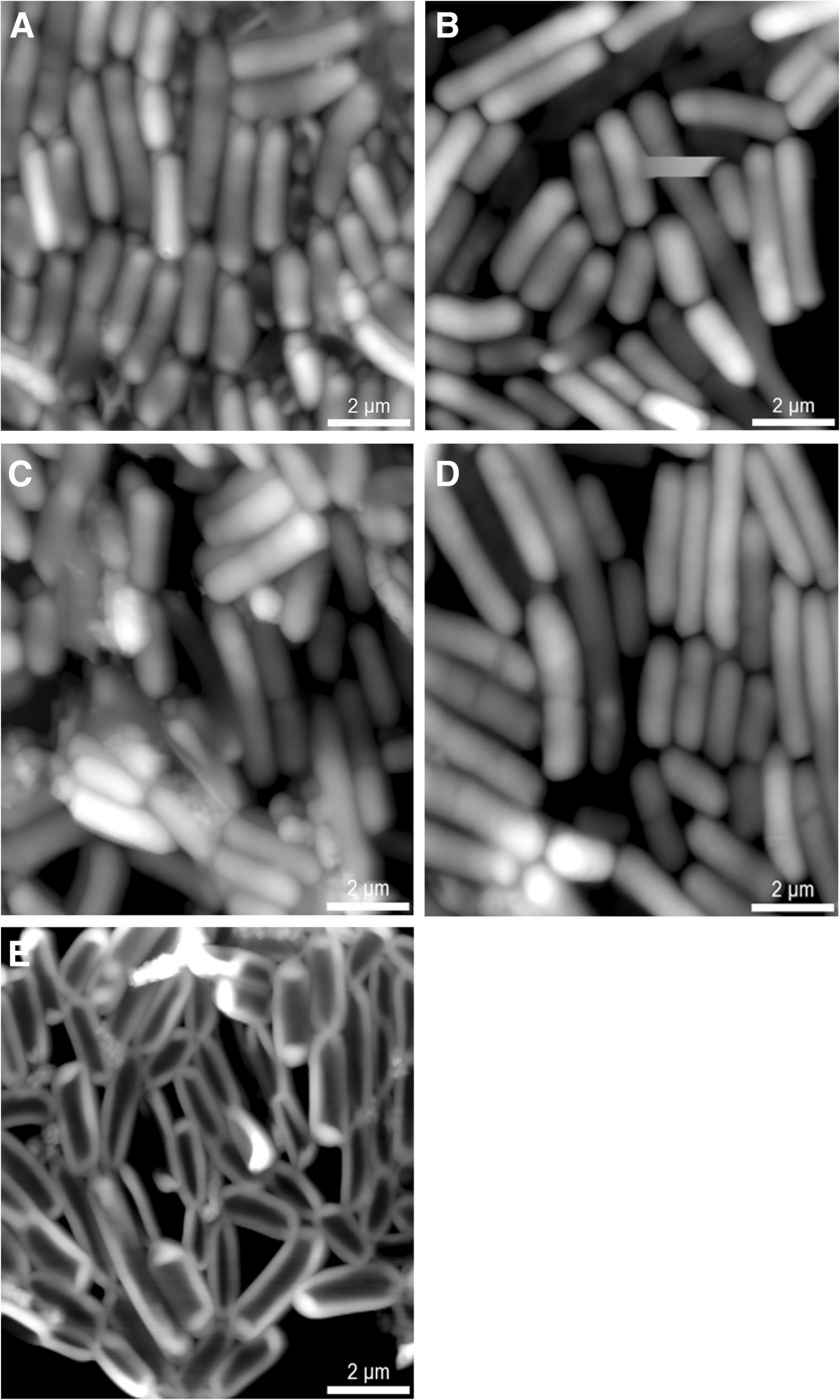
Contains illustrations of AFM height monolayer maps of bacteria on the mica substrate. **a** *B. animalіs* VKL. **b** *B. animalіs* VKВ. **c** *L. acidophilus* ІМV В-7279. **d** *L. casei* ІМV В-7280. **e** *L. delbrueckii* subsp. *bulgaricus* ІМV В-7281

AFM maps of bacteria monolayer heights on the mica substrate are shown in Fig. 1. It was established that the excess moisture removal did not cause the destruction of *L. acidophilus* IMV B-7279, *L. casei* IMV B-7280, *B. animalis* VKL, and *B. animalis* VKB, so it was possible to estimate their cell wall elasticity, without resorting to a more painstaking measurements in liquid buffer. The exception was *L. delbrueckii* subsp. *bulgaricus* IMV B-7281 strain, which’s cell wall after the excess moisture removal sank under its weight. This is probably an indication of its low elasticity compared with other strains that we studied.

Since the image in the AFM is formed on the basis of the control of force interaction of the solid-state probe with the bacteria surface, there is an opportunity not only to diagnose the surface but also to modify it. By means of checking if it is possible to get information about the peculiarities of the power interactions in a given point of the surface by registering the dependence of the console probe deformation on the tip-surface distance. Application of the controlled force to the probe, which is in contact with the much softer surface, causes its elastic–plastic deformation. Under these conditions force spectroscopy is similar to *nanoindentation* for the mechanical properties study of surfaces at the nanoscale [46].

The recorded data in the central parts of the bacteria in the field of loads 0.5–3.0 mcN clearly shows a linear plot, which corresponds to the depth of the AFM edge probe penetration into a bacterium by 5–25 nm. The slope of the plot quite well characterizes the elasticity of the probe-wall contact. Therefore, to obtain the relative elasticity characteristics of the probe-wall contact, histograms of the slope coefficient distribution of the line (the rigidity of the contact) were built.

**Fig. 2:**
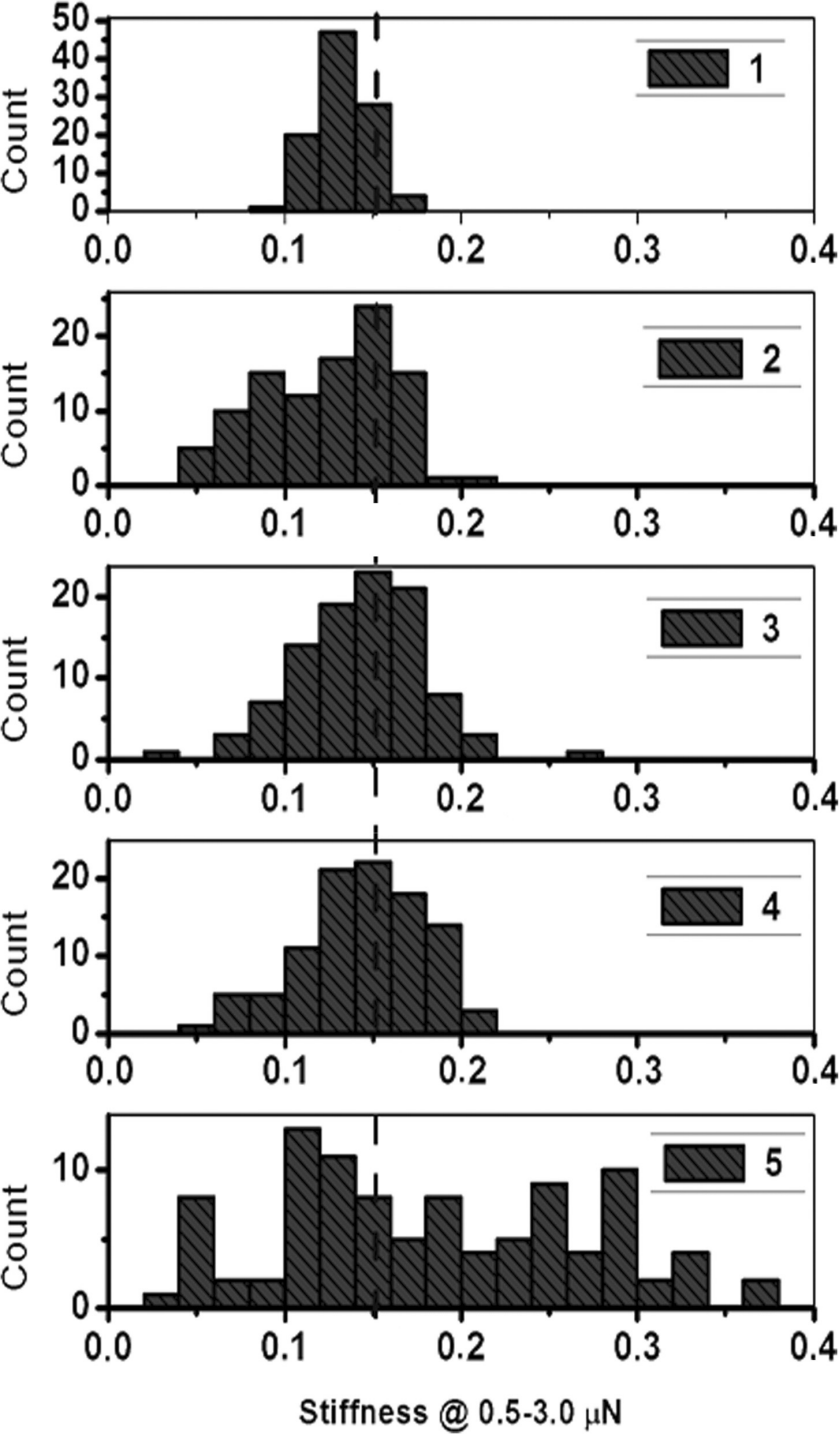
Histograms of the cell elasticity distribution of LAB and bifidobacteria: *1 Bifidobacterium animalis* VKL, *2 Bifidobacterium animalis* VKB, *3 Lactobacillus acidophilus* ІМV В-7279, *4 Lactobacillus casei* ІМV В-7280, *5 Lactobacillus delbrueckii* subsp*. bulgaricus* ІМV В-7281

According to the measurements, the histograms of the cell wall’s elasticity (rigidity) distribution of the bacteria, which were investigated, were built (Fig. 2). It was established that the rigidity of the cell walls among lactobacilli was distributed as follows: *Lactobacillus acidophilus* IMV B-7279 > *Lactobacillus casei* IMV B-7280 > *Lactobacillus delbrueckii* subsp. *bulgaricus* IMV B-7281. Among the strains of bifidobacteria, the most rigid cell wall was in *B. animalis* VKB in comparison with *B. animalis* VKL.

Among the lactobacilli, the most elastic cell wall was found in *L. delbru*е*ckii* subsp. *bulgaricus* IMV B-7281 and among bifidobacteria—in *B. animalis* VKL.

In further steps of our research, we established that the strains of lactic acid bacteria and bifidobacteria with varying degrees of cell wall rigidity had different survival after their joint cultivation with macrophages throughout 90 and 120 min (Table 1). We have not observed a significant difference between the number of CFU of lacto- and bifidobacteria that were survived after 30 and 60 min of cultivation with macrophage lysates. However, after 90 and 120 min of *L. acidophilus* IMV B-7279 and *L. casei* IMV B-7280 incubation with macrophages, this strains survived better than *L. delbru*е*ckii* subsp. *bulgaricus* IMV B-7281 that had a more elastic cell wall according to the AFM data. As can be seen from the data presented in Table 1, *B. animalis* VKL with a more elastic cell wall according to the AFM data also survived in much smaller numbers after cultivation with macrophages during 90 and 120 min than *B. animalis* VKB. After 24 h of lactic acid bacteria and bifidobacteria cultivation with macrophages lysates, all the strains did not survive. That is, lacto- and bifidobacteria survival in the macrophages depended not only on the elasticity of bacterial cell wall but also on the time of their joint cultivation.

**Table 1:** The number of LAB and bifidobacteria that were seeded out from macrophage lysates

Strains	Number of CFU/time of cultivation			
	30 min	60 min	90 min	120 min
*B. animalis* VKL	58 ± 5	120 ± 6	100 ± 4*	43 ± 3*
*B. animalis* VKB	61 ± 3	135 ± 6	250 ± 7	107 ± 5
*L. acidophilus* IMV В-7279	43 ± 3	97 ± 3	120 ± 5	90 ± 4
*L. casei* IMV В-7280	74 ± 4*	105 ± 5	82 ± 3*	41 ± 3*
*L. delbruеckii* subsp*. bulgaricus* IMV В-7281	45 ± 3	83 ± 4*	47 ± 2*	2 ± 1*

^*^ *P* < 0.05 in comparison with the indicators for the strains of the same genus

Strains of lactic acid bacteria and bifidobacteria in vitro had a stimulating effect on the functional activity of macrophages. There was an increase in their ability to produce NO/NO_2_, which was proved by the increase of its concentration in the cultural medium after 24 h joint peritoneal exudate macrophage cultivation with these strains. The data obtained in the experiment are given in Table 2. Among the strains of bifidobacteria, which we examined, the most stimulated effect on the NO/NO_2_ production had bacteria with more rigid cell wall—*B. animalis* VKB in comparison with *B. animalis* VKL.

**Table 2:** The level of NO/NO_2_ in macrophage supernatants after their joint cultivation with LAB and bifidobacteria

Group	Optical density of the sample	Macrophage activation index, T
	λ = 520 nm	%
Macrophages that were cultivated without bacteria	0.071 ± 0.005	–
Macrophages that were cultivated with *B. animalis* VKL	0.133 ± 0.009*	89.13
Macrophages that were cultivated with *B. animalis* VKB	0.146 ± 0.014*	106.77
Macrophages that were cultivated with *L. acidophilus* IMV В-7279	0.161 ± 0.013*	128.82
Macrophages that were cultivated with *L. casei* IMV В-7280	0.161 ± 0.015*	128.82
Macrophages that were cultivated with *L. delbruеckii* subsp*. bulgaricus* IMV В-7281	0.197 ± 0.007*	179.21

*P < 0.05 in relation to indicators for intact mice

Among lactobacillus strains, the ability to increase the nitric oxide production by peritoneal exudate macrophages was distributed as follows: *L. delbrueckii* subsp. *bulgaricus* IMV B-7281 > *L. acidophilus* IMV B-7279/*L. casei* IMV B-7280. That is, *L. delbrueckii* subsp. *bulgaricus* IMV B-7281 with a more elastic cell wall, according to the AFM data, intensified actively NO/NO_2_ production by macrophages in comparison with other strains of lactobacilli, which we studied. The obtained data showed that the most effective inductors of NO/NO_2_ production by macrophages among the strains of bifidobacteria was *B. animalis* VKB with a more rigid cell wall, and among the lactobacillus strains—*L. delbrueckii* subsp. *bulgaricus* IMV B-7281 with a more elastic cell wall.

The ability of macrophages to accumulate the reactive oxygen metabolites in vitro also increased under the influence of lactic acid bacteria and bifidobacteria strains, which we examined (Table 3). Lactic acid bacteria strains’ ability to activate oxygen-dependent bactericidal activity of macrophages can be divided as follows: *L. delbru*е*ckii* subsp. *bulgaricus* IMV B-7281 > *L. acidophilus* IMV B-7279 > *L. casei* IMV B-7280. Thus, *L. delbru*е*ckii* subsp. *bulgaricus* IMV B-7281 with more elastic cell wall increased more effectively oxygen-dependent bactericidal activity of macrophages than other strains of lactic acid bacteria with more rigid cell wall. Along with this, *B. animalis* VKL and *B. animalis* VKB (each strain separately) activated oxygen-dependent bactericidal activity of macrophages with equal efficiency.

**Table 3:** The influence of LAB and bifidobacteria strains to the ability of peritoneal exudate macrophages to accumulate the reactive oxygen compounds

Group	Optical density of the sample	Index of peritoneal exudate macrophage activation
	(λ = 492 nm)	(%)
Macrophages that were cultivated without bacteria	0.069 ± 0.002	–
Macrophages that were cultivated with *B. animalis* VKL	0.134 ± 0.014*	94.52*
Macrophages that were cultivated with *B. animalis* VKB	0.137 ± 0.003*	98.55*
Macrophages that were cultivated with *L. acidophilus* IMV В-7279	0.123 ± 0.004*	78.26*
Macrophages that were cultivated with *L. casei* IMV В-7280	0.100 ± 0.005*	44.28*
Macrophages that were cultivated with *L. delbruеckii* subsp*. bulgaricus* IMV В-7281	0.134 ± 0.002*	93.88*

*P < 0.05 in relation to indicators for intact mice

It was found that lactobacilli strains in vitro increased the production of IL-12 and IFN-γ in the macrophage culture (Table 4). However, IL-12 titers were higher in the macrophages culture that were cultivated with *L. acidophilus* IMV B-7279 or *L. casei* IMV B-7280 (each strain separately) than with *L. delbru*е*ckii* subsp. *bulgaricus* IMV B-7281. Production of IFN-γ more efficiently increased under the influence of *L. casei* IMV B-7280 as compared with other strains of lactobacilli, which we studied. *B. animalis* VKL and *B. animalis* VKB strains did not affect the level of these cytokine production by macrophages.

**Table 4:** The concentration of immunoregulatory cytokines—IFN-γ and IL-12 in supernatants from the cultivation suspension of peritoneal exudate macrophages with probiotic strains of LAB and bifidobacteria

Group	ІFN-γ concentration	ІL-12 concentration
	(pg/ml)	(pg/ml)
Macrophages that were cultivated without bacteria	60.2 ± 1.1	11.0 ± 0.5
Macrophages that were cultivated with *B. animalis* VKL	72.3 ± 2.4	5.0 ± 0.2
Macrophages that were cultivated with *B. animalis* VKB	77.7 ± 4.0	3.0 ± 0.4
Macrophages that were cultivated with *L. acidophilus* ІМV В-7279	86.2 ± 2.5*	200.6 ± 16.7*
Macrophages that were cultivated with *L. casei* ІМV В-7280	133.2 ± 7.1*	263.6 ± 20.6*
Macrophages that were cultivated with *L. delbruеckii* subsp*. bulgaricus* IMV В-7281	80.6 ± 2.3*	99.0 ± 10.3*

*P < 0.05 in relation to indicators for intact mice

## Discussion

Our results demonstrated that *L. delbru*е*ckii* subsp. *bulgaricus* IMV B-7281 strain with more elastic cell wall was more effectively digested in the macrophages and had better activating action on the functional activity of macrophages in vitro, nitric oxide production, and their ability to accumulate reactive oxygen metabolites than *L. casei* IMV B-7280 and *L. acidophilus* IMV B-7279 strains with more rigid cell walls. Probably, bacterial derivatives, which formed during cell’s digestion in the macrophages, are important for the activation of oxygen-dependent bactericidal activity of macrophages and their ability to produce nitric oxide under the influence of lactobacilli.

On the other hand, the activation of IL-12 production was more effective in the case of macrophage cultivation with *L. casei* IMV B-7280 or *L. acidophilus* IMV B-7279 with more rigid cell wall compared with *L. delbru*е*ckii* subsp. *bulgaricus* IMV B-7281. *L. casei* IMV B-7280 more efficiently induced IFN-γ production.

Thus, bacterial strains with more rigid cell wall according to AFM data were more effective inductors of cytokines in the macrophages culture than the strains with more elastic cell wall.

This may indicate the fact that interaction between the components of the integrated cell walls of lactobacilli with TLR- and/or NOD-receptor is necessary for the induction of cytokine production and the subsequent activation of macrophages.

However, strains with more elastic cell wall effectively activated effector functions of macrophages. So, it is possible to assume that combination of strains with different degrees of cell wall elasticity in one composition will be useful to create drug with immune-modulatory activity for immune system correction in various pathological conditions.

At the same time, other data were obtained in the study of the bifidobacteria strains’ influence on the functional activity of macrophages in vitro. *B. animalis* VKB, which was more resistant to the intracellular digestion in the macrophages, increased their oxygen-dependent bactericidal activity like *B. animalis* VKL with a less rigid cell wall. Both strains of bifidobacteria did not influence the IL-12 formation in the macrophage culture.

In previous studies, we have noticed the ability of the studied strains of lactic acid bacteria and bifidobacteria to activate peritoneal exudate macrophages of mice [47]. *L. bulgaricus* CRL 423 [48], *L. casei*, and *L. bulgaricus* LB 51 [49, 50] strains had similar impact on the phagocytic activity of macrophages.

According to the results of the present study, we have observed the dependence of the cell wall elasticity of lactic acid bacteria and bifidobacteria and their ability to activate monocyte–macrophage link of the immune system, which is confirmed by the results of the research by *Schär-Zammaretti* et al. [51]. It was determined that probiotic strains of microorganisms with a more rigid cell wall maintained its viability in the macrophages for a longer period of time. So, their interaction with Toll-like and NOD-like receptors was longer and the activation of macrophages was more significant.

All the investigated strains of lactic acid bacteria and bifidobacteria significantly activated the metabolic activity of peritoneal exudate macrophages after their joint cultivation. We have noted the relationship between the cell wall rigidity and the degree of macrophage activation. At the same time, *L. delbru*е*ckii* subsp. *bulgaricus* IMV B-7281, that had the most elastic cell wall, caused the considerable activation of the phagocytes, but, according to the literature [22] and our pilot observations (*unpublished data*), this strain was able to synthesize significant amounts of exopolysaccharide, which shows good immune-modulatory properties including the ability of mononuclear phagocytes activation [52–55].

It is known that *nitrosative stress* via the formation of nitric oxide together with the formation of reactogenic oxygen compounds by phagocytic system cells is one of the main mechanisms of antimicrobial macroorganism protection. The best stimulators of nitric oxide by macrophages were strains with more rigid cell wall [56]. Similar data regarding the stimulating effect on the nitric oxide formation by peritoneal exudate macrophages in their joint cultivation with heat- inactivated strains of *Bifidobacterium*, *Lactobacillus acidophilus*, *L. bulgaricus*, *L. casei*, *L. gasseri*, *L. helveticus*, *L. reuteri* and *Streptococcus thermophilus* or cell walls components were also obtained by other researchers [56].

According to the patterns of cytokine induction that were described in the literature, some strains of lactic acid bacteria can stimulate macrophages and dendritic cells to the IL-12 synthesis [38, 54, 56], which, along with IFN-γ, play a key role in the activation of cell-mediated immunity. All the investigated bacterial strains stimulated significantly peritoneal exudate macrophages to the IL-12 production. Phagocytosis of lactic acid bacteria was necessary for the induction of this cytokine, and strains with more rigid cell wall, that were more resistant to the action of macrophages lytic enzymes, were the best inductors of IL-12.

All the studied strains had high interferonogenic properties. In previous experiments, we noticed the ability of *Lactobacillus casei* IMV B-7280 and *Lactobacillus acidophilus* IMV B-7279 to enhance the level of interferon synthesis after oral administration in the in vivo experiments [54, 57]. These strains appeared to be the most active in the interferonogenesis stimulation on the model of peritoneal macrophages in vitro. It should be noted that bifidobacteria induced IFN-γ production less than lactobacilli.

Thus, our data demonstrated close relationships between the cell wall elasticity of lactic acid bacteria, their resistance to the intracellular digestion in macrophages, and the ability to increase macrophages functional activity.

### Consolidation of the PPPM concept

Our research result data can be easily applied in the clinical setting; propose the improvement of selection of the of probiotic strains with potentially beneficial properties via estimation of the cell wall elasticity, in regard to the immune-modulatory properties of the strain, will help expand the range and quality of probiotic products available on the market, as an in vitro study significantly saves time and allows to cut-off unpromising strains in the early stages (predictive).

Consequently, a research technique for cell wall properties like elasticity can be used for massive screening to find strains of probiotic bacteria promising to have a highly specific effect on the immune system (as the subject of personalization), since we have demonstrated that the elasticity of the cell wall is linked with the speed of digestion microorganism in macrophages, respectively, with the strength and direction of its effects on the host immune system.

For preventive medicine, in those cases, when we again need to achieve a certain effect—some improvement of the microflora as prophylaxis, but additional stimulation of immunity is not necessary. In this case, the primary method of screening strains by determining the elasticity of cell wall allows to select probiotic bacteria that do not have immune-modulatory effect and will not stimulate the immune system.

### Limitations of the study and bottlenecks for the follow-up research

It was an in vitro study where the survey was conducted on mice macrophage culture. The study itself did not suppose any particular immune-related pathology on animal model and in humans.

We have observed low expected results in evaluation of cell wall elasticity via AFM, considering that peptidoglycan, as immune response stimulating component of the cell wall, is known also as a wall elasticity-inducing factor. This non obvious finding might be explained by considerable variation in the cell surface properties, environmental stressors affecting the cell surface architecture by influencing PG biosynthesis, expression of EPS, and cell surface proteins that are important for the adaptations of bacteria during functioning and survival of these bacteria in the GIT [21]; and also the fact that it was assessed by AFM scanning, and in the same time provide promising insights for future research. These results, contributing to current knowledge and plans of further research of the molecular mechanisms underlying the physiological characteristics of probiotic cells, however, need further validation.

Immune response mechanisms including TLR and pathogen-associated molecular patterns (PAMPs), as molecules associated with groups of pathogens, recognized by cells of the innate immune system and negative regulators could have an important physiological impact on maintaining or re-establishing homeostatic signals [58]. This mechanism was not considered and needs further research. Identification and validation of effector molecules complemented with parallel studies for their relevant receptors in the host cells can strengthen the strain specificity and contributes to the development of probiotics with enhanced health benefits [21]. Age- and gender-related studies are necessary, as You et al. found that upon pretreatment of young or old dendritic cells (DCs) with LPS or probiotics, proliferation of T cells derived from older donors was not enhanced [59].

Survival of probiotics inside the phagosome also has not been investigated. However, both live and dead cells of probiotic bacteria can evoke immune response and generate beneficial biological responses. This has several implications for the production and application of probiotics, as it will be difficult to assess the relative proportions of live and dead cells in a probiotic culture. A major problem in the practical application of probiotics and also in the understanding of their mode of action is that heterogeneous effects are often obtained [60]. Live probiotic cells influence both the gastrointestinal microflora and the immune response while the components of dead cells exert an anti-inflammatory response in the gastrointestinal tract.

The study of effects of live and dead bacteria and their most beneficial proportions, considering application of lysates in particular for immune-related diseases, is an important issue for further research. This would involve investigating the potential for novel probiotic bacteria via development from fecal microbiota and improvement of existing industrial probiotic bacteria strains using techniques like polymerase chain reaction denaturing gradient gel electrophoresis (PCR-DGGE) analysis and AFM data, phenotypes associated with diverse hydrolases [31], PAMPs, MAMPS, receptors, use of recombinant DNA technologies like recombineering [26], and other relevant methods.

Larger studies of the cell wall are needed that should aim to study the correlations among cell wall elasticity, cell components, AFM data, genetic sequencing, and mutant analyses data in regard to particular cell wall component synthesis for the identification of several proper effector molecules of probiotic cell wall responsible for the most beneficial treatment effect.

It is recommended to consider development of extensive probiotic profiles in regard to those based on studies of parameters to identify mechanism of action in vivo, followed by clinically relevant steps, according to guidelines [61]. The probiotic strain profiles should allow differentiation of experimental groups providing implementation of screening for potential probiotic strains based on their properties with fast translation.

In this matter, the primary method of screening might be the determination of cell wall elasticity via AFM, which may enable selection of probiotic bacteria that do not have immune-modulatory effects and will not stimulate the immune system.

Animal model studies are suggested for specific immune-related processes (autoimmunity, asthma, diabetes, gout, hepatitis, cancer, CVD, etc.), based on beneficial bacteria application as foods and colonic microbiota transplantation using hybrid designs, including gnotobiotic and the Simulator of Human Intestinal Microbial Ecosystem (SHIME^1^) application as a dynamic and controlled model of the gastrointestinal tract [62].

Development of sophisticated microbiome and microbe-host interaction imaging technique in vivo, including radiology, endoscopy, microscopy data—as exemplified here by AFM—may be a basis for developing hybrid techniques. These would include tools for performing surgical operations on living cells at nanoscale resolution, e.g., using AFM and scanning confocal microscopy [63], which can directly reveal the needle position, penetrated both the cellular and nuclear membranes to reach the nucleus. This technique enables the extended application of AFM to analyses and surgery of living cells. The potential alternative for probiotics might be the suggested lysates of probiotic strains that also exhibit immune-modulatory activity.

Studies on relevant dietary components, including prebiotics and fermented foods, and development of novel probiotic-based personalized diets and specific biological treatments (as FMT) are required via the development and application of products manufactured from one’s own probiotic strains of organism, considered promising to achieve the personalized approach.

Promising next steps for research include studies on the extra-colonic microbiome in the oral cavity, nasopharynx, respiratory tract, vagina, endometrium, bladder and skin, as well as on the effects of probiotic interventions using proven and newly developed strains to benefit relevant microbiome correction, in particular for allergies, infections, injuries, wound healing, and cancer.

While developing the concept, we should keep in mind development of novel electronic tools, including medical gadgets to guide microbiome improvement and enable feedback-based diets [64].

We still note a lack of radiology and endoscopy imaging modalities to visualize human and laboratory animal microbiome in vivo.

Imaging in vivo techniques, such as positron emission tomography (PET), allow the determination of both the location and the number of infection and inflammatory foci in virtually all tissues, may offer promising perspectives e.g., for the future application of [18F]UBI 29–41 as a PET-imaging ligand.

In order to determine if this compound will be useful for in vivo discrimination between bacterial infections and sterile inflammatory processes, more studies have to be considered [65, 66].

Non-invasive techniques, such as *CT*, *MRI*, and *US* may support additional information regarding colonic microbiota and the colonic mucosa; muscles and nerves continue microbiota-related inflammatory morphologic changes of tissues particular in the colon. Thus US specifically enables detection and quantification of inflammation in a murine acute colitis model; US imaging with MBSelectin correlates well with FDG uptake at PET/CT imaging [66].

Karunatilaka et al. [67] used nanometer-scale superresolution imaging to reveal dynamic interactions between the proteins involved in starch utilization system (Sus) complex assembly and function during glycan catabolism in human gut Bacteroidetes in real time in live cells.

In vivo *molecular imaging* systems for animals have become indispensable research tools in many clinical and basic research laboratories. But developers are now pushing the technology further in the hopes of making a new generation of platforms with greater accuracy and sensitivity for a wider array of applications. Bioluminescence and fluorescence actually give us the opportunity to target particular cellular processes occurring in vivo [68].

Intestinal *[18F]FDG PET* is a reliable method for the estimation of intestinal glucose uptake (GU). Thus, imaging data from animal and human studies by Honka et al. provided evidence regarding insulin sensitivity in the mucosal layer of the intestine and of intestinal insulin resistance in obese non-diabetic human participants [69]. The occurrence of mucosal insulin resistance in the early stages of the metabolic syndrome may partly be a cause or an effect of the typical changes in intestinal function observed in these illnesses, most notably altered incretin production [70]. Metabolic endotoxaemia and gut-bacterial interaction [70], probiotic cell wall ingredients might be a source of potential biomarkers for molecular imaging.

## Conclusions

We have established the following:

- ▪ The effect of the living cultures of lactic acid bacteria on activation of monocyte–macrophage link of immunity in the experiments in vitro was detected.
- ▪ The strains of lactic acid bacteria and bifidobacteria in vitro had a stimulating effect on the functional activity of macrophages. There was an increase in their ability to produce NO/NO_2_.
- ▪ There is a close relationship between the elasticity of the cell wall of lactobacteria, their resistance to the intracellular digestion in macrophages, and the ability to increase macrophages functional activity.
- ▪ The joint cultivation of investigated strains (alone) with macrophages of peritoneal exudate caused their activation and stimulation of immune-modulatory cytokines and active oxygen and nitrogen oxide compound production.
- ▪ The combination of strains with different degrees of cell wall rigidity in one composition will be useful to create a drug with immune-modulatory activity for immune system correction in various pathological conditions.

### Expert recommendations

Further studies including sufficient cohorts of volunteers are necessary to translate model data of probiotic efficacy to human population as well as conduction of epidemiological studies for obtaining evidence regarding adverse effect of particular food additives. In addition, it is needed to study microbiota on relevant models related to specific processes (autoimmunity, asthma, diabetes, gout, hepatitis, cancer, NDD, CVD, thyroid, rheumatic diseases, etc.). Obtained results may indicate the effectiveness of probiotic therapy to prevention in human based on personalized microbiota additives. To achieve the personalized approach, the development and application products manufactured from own strains of organisms are considered promising. The potential alternative for probiotics might be the suggested lysates of probiotic strains that also exhibit immune-modulatory activity.

We also suggest the further studies regarding dietary additives, immune and inflammatory responses, and develop and test promising nanomaterials for prospect of complex multiparametric impact to gastrointestinal microbiota, gut–brain axis.

After approval, development of safe and effective person-related medications and dietary additives with future clinical testing should be initiated to implement results for routine practice.

*As the concluding points*, we can formulate the following proposals (expert recommendations):

1. For the European Union (EU):
  ▪ create an international project(s) to study the effects of probiotics for the implementation of evidence-based personalized dietology for prevention of a wide scope of diseases for promotion of health within inclusive patient profiling and in the integrated vision of *interactome*;
  ▪ to support and promote integrative agreed position for development biological therapies under EPMA umbrella, to increase their evidence, study adverse effects, and popularize safe and effective approaches;
  ▪ to agree with national (including associated countries) and EU nutritional standards for food compositions and probiotic databases and development evidence-based guidelines;
  ▪ to study the immune-modulatory mechanisms of probiotics for prevention and treatment of lung disease and asthma, diabetes, gout, hepatitis, NDD, CVD, thyroid, rheumatic diseases, and cancer in multidisciplinary scope considering many factors, including life style, smoking, and other forms of tobacco consumption, sport, stress, emotions, psyche, sleep disorders, etc.
2. For national level (in Ukraine):
  ▪ to create and promote a consolidated Nutrition Association under EPMA umbrella to improve coordination and reduce the overlap between national and EU funding in relevant fields of research;
  ▪ to mobilize SMEs, when appropriate, in the transnational projects to enable fast implementation of innovative solutions;
  ▪ to participate in the projects in partnership with EU to follow up experimental and clinical trials and involve related institutions and centers to the study.

## Abbreviations

PPPM: predictive, preventive, and personalized medicine
LAB: lactic acid bacteria
AFM: atomic force microscopy
DCs: dendritic cells
GBA: gut–brain axis
PRRs: pattern-recognition receptors
NLRs: NOD-like receptors, nucleotide-binding oligomerization domain receptors
TLR: Toll-like receptors
PG: peptidoglycan
EPS: exopolysaccharides
LPS: lipopolysaccharide
DGGE: denaturing gradient gel electrophoresis
FMT: fecal microbiota transplantation
CVD: cardiovascular diseases
T2D: diabetes mellitus type 2
NDD: neurodegenerative diseases

## Footnotes

1 SHIME is a registered trademark of ProDigest (Ghent, Belgium)
